# Post-Cueing Deficits with Maintained Cueing Benefits in Patients with Parkinson’s Disease Dementia

**DOI:** 10.3389/fneur.2014.00236

**Published:** 2014-11-17

**Authors:** Susanne Gräber, Inga Liepelt-Scarfone, Ilona Csoti, Walter Maetzler, Fahad Sultan, Daniela Berg

**Affiliations:** ^1^Department of Neurodegeneration, Hertie Institute for Clinical Brain Research, University of Tübingen, Tübingen, Germany; ^2^German Center of Neurodegenerative Diseases (DZNE), Bonn, Germany; ^3^Department of Neurology, Gertrudis Hospital, Leun-Biskirchen, Germany; ^4^Department of Cognitive Neurology, Hertie Institute for Clinical Brain Research, University of Tübingen, Tübingen, Germany

**Keywords:** Parkinson’s disease, dementia, external cueing, reaction time, non-pharmacological therapy, basal ganglia, permanent cueing

## Abstract

In Parkinson’s disease (PD), internal cueing mechanisms are impaired leading to symptoms like hypokinesia. However, external cues can improve movement execution by using cortical resources. These cortical processes can be affected by cognitive decline in dementia. It is still unclear how dementia in PD influences external cueing. We investigated a group of 25 PD patients with dementia (PDD) and 25 non-demented PD patients (PDnD) matched by age, sex, and disease duration in a simple reaction time task using an additional acoustic cue. PDD patients benefited from the additional cue in similar magnitude as did PDnD patients. However, withdrawal of the cue led to a significantly increased reaction time in the PDD group compared to the PDnD patients. Our results indicate that even PDD patients can benefit from strategies using external cue presentation but the process of cognitive worsening can reduce the effect when cues are withdrawn.

## Introduction

Parkinson’s disease (PD) is defined as a neurodegenerative disorder affecting nigrostriatal neurons in the basal ganglia (BG) circuit. The BG are involved in different processes related to motor function or other non-motor abilities, such as cognitive performance. They play a key role in generating and monitoring motor programs especially those responsible for the “automatic execution of learned motor plans” (1). Moreover, the BG cue the end of a preparatory activation or a preceding sub-movement in the supplementary motor area (SMA) to give way to initialize a new component of a movement sequence (2).

In PD, the impairment of the BG-triggered internal cue results in the well-known cardinal symptom of hypokinesia (3).

Presentation of an external cue can reduce motor problems (e.g., gait variability) in PD. This may be due to improved allocation of attentional resources (3–6). In contrast, internal cues (attentional strategies) can increase motor problems (7). The positive effect of an external cue can be introduced with either visual or auditory presented cues (8). Motor activity, triggered by sensory input does not necessarily depend on BG involvement. A study with PD patients revealed that damaged BG can be bypassed by visual input through neuronal circuits involving cerebro–cerebellar–cerebral pathways resulting in improved movement abilities in response to the stimulus (9). Additionally, cortical activity increases when triggered by an external cue and thus can dampen or even suppress the pathological BG activity in PD to facilitate movement (10, 11). The fact that external cues help to suppress and/or bypass the BG implies that in addition to the participation of motor areas, sensory and higher cortical areas are also involved in movement initiation. This may even include prefrontal regions (12) known for their involvement in higher cognitive functions, such as executive functions and attention, which are also known to be vulnerable in the dementing process in PD (13).

The influence of external cues on complex motor performance, such as gait has been intensely studied. Moreover, the addition of external cues may not always be beneficial as they represent a dual task with the probability to distract the affected person and, therefore, can lead to dangerous situations. This may be particularly relevant for patients with impaired cognition as they have an increased probability to suffer from deficits in attention, prioritization (14), or executive functions as shown in a study investigating patients with Alzheimer’s disease and rhythmical auditory cueing (15). The additional stimuli produced deleterious effects on gait. However, the effect of external cues in PD patients having a substantial cognitive deficit is not well understood. Nearly 80% of PD patients develop Parkinson’s disease dementia (PDD) as a prominent sign of the progressive neurodegenerative process (16). In PDD, Lewy body pathology affects the BG region and also involves higher cortical areas (17).

The aim of this project was to investigate the impact of a substantial cognitive deficit in PD on the response to external cues during and after the cue has been presented. Therefore, we compared the performance of PDD and non-demented PD patients (PDnD) in a simple reaction time (SRT) task.

## Materials and Methods

### Subjects

From a cohort of total 121 PD patients, including 34 patients with the clinical diagnosis of PDD (Movement Disorders Society Task Force, the consensus guideline; see below), we selected a group of 25 PDD patients and 25 PDnD patients who completed the reaction time task described below according to best match in age and gender. For our primary criterion age, we chose the participants by matching them individually for a best correspondence in age ±3 years. Therefore, nine participants from the original group of 34 PDD patients had to be excluded due to advanced age. In a second step, we selected participants to obtain two groups similar in gender resulting in the two groups of 25 participants each (see Table 1). The cohort of 121 patients with PD was recruited from the outpatient clinic of the Department for Neurodegenerative Diseases at the University of Tübingen and the Gertrudis Clinic Leun-Biskirchen, both in Germany. The neuropsychological data from all patients of this cohort have been published previously in an article focusing on cognitive profiles in PD and their relation to dementia (18). Diagnosis of PD was made according to the UK Brain Bank criteria. All participants were German native speakers and had normal or corrected hearing and visual abilities. All assessments were carried out on patient’s optimized dopaminergic medication. Exclusion criteria were history of other neurological diseases affecting the central nervous system, deep brain stimulation, or diagnosis of mild cognitive impairment (19). The study was approved by the local ethical committee. All participants gave written informed consent.

**Table 1:** **Demographic characteristics of the two study groups: Parkinson’s disease with dementia (PDD) and PD non-demented (PDnD)**.

	PDD	PDnD	*p*-value
*n*	25	25	–
Sex (m)	17	20	0.520
Age	72.52 (3.86)	71.32 (3.13)	0.234
Disease duration	9.91 (4.7)	7.8 (5.62)	0.156

### Simple reaction time task

To assess the use and the withdrawal of an external cue, a SRT task was used. This task was taken from an established test battery for clinical and experimental examination of different aspects of attention [subtest “Alertness” from test for attentional performance (TAP)] (20). The participant had to press a button in front of her/him on the table with the right index finger as soon as a visual target (“X”) appeared. The “X” was presented in the center of the computer screen with a size of 2°18′ degrees of visual angle according to the test manual (21, 22). The task was displayed on a 15 in screen with the participant sitting in a comfortable position at an average distance of 60 cm from the screen. The stimulus was presented under two different conditions (A and B) resulting in four blocks (ABBA) of 20 trials each. In condition A (block 1 and block 4), only the visual target appeared.

In condition B (block 2 and block 3), an additional acoustic cue (warning tone) was administered 600–1500 ms before the visual stimulus to speed up reaction time (22). The acoustic signal was a single tone with a frequency of 1000 Hz and a duration of 400 ms. The tone was presented in a comfortable loudness to the participant and was adjusted as needed in a test phase before the performance of the experiment. Intervals between the reaction and the next stimulus or next warning tone were set in the range of 1800– 2700 ms. The respective blocks were presented according to the test protocol with time intervals of about 2–10 s between the blocks.

The reaction time (in ms) of each single trial was recorded and considered for data analysis. In measuring reaction time using the median shows clear advantage reducing possible influence of outliers (potentially caused by artifacts) on the data. For each parameter, the medians were calculated and group differences were analyzed using the means of the medians (Mm). To evaluate time differences referring to the transitions between different blocks and conditions, the parameter TbB (transition between blocks) was defined as “Mm of block x + 1 − Mm of block x” resulting in the three parameters TbB1 (Mm block 2 − Mm block 1), TbB2 (Mm block 3 − Mm block 2), and TbB3 (Mm block 4 − Mm block 3). Positive values of the TbB indicate a longer reaction time in the following condition in relation to the preceding condition. Negative values of the TbB represent a shorter reaction time in the following condition in relation to the preceding condition. Higher values (positive or negative) indicate a greater contrast in reaction time between the adjacent blocks. To exclude the possibility of systematic variability on reaction time within the respective blocks due to a progression of reaction time shortening by repeated trials of the same type, we conducted an ANOVA with repeated measurement comparing the median of the first five trials (Trails 1–5) with the median of the last five trials (Trails 16–20) of each block (1–4) with main factor group of participants (PDD or PDnD).

### Neurological assessment

Neurological assessment included the motor part of the Unified Parkinson Disease Rating Scale [UPDRS III (23)].

### Diagnosis of dementia

Diagnostic criteria for dementia (PDD) were in accordance with the Clinical diagnostic criteria of the Movement Disorders Society task force for probable PDD (13, 24). Criteria for PDD were (1) a test performance of 1.5 standard deviation (SD) below the mean normative data in at least two of the defined cognitive domains (attention, executive functions, visuospatial function, memory, language abilities), plus (2) a history of cognitive decline with insidious onset and slow progression, plus (3) reported impairments in non-motor activities of daily living by patients and/or their caregivers. To estimate the performance in the relevant cognitive domains, the results of the comprehensive neuropsychological test were used. These are described in the previously published paper (18).

### Statistics

SPSS 20.0 for Windows (SPSS Inc., Chicago, IL, USA) was used for statistical analysis. Descriptive data were presented as mean (M) and SD for the demographic parameters and as mean of medians (Mm) and SD for the parameters related with reaction time.

Demographic and clinical data between the groups (PDD and PDnD) were compared using either the Student’s *t*-test or an analysis of covariance (ANCOVA) or repeated measurement ANOVA (analysis of variance). To eliminate possible motoric influences on the data, we conducted an ANCOVA corrected by the UPDRS motor scale.

## Results

### Total reaction time (all conditions)

**Figure 1:**
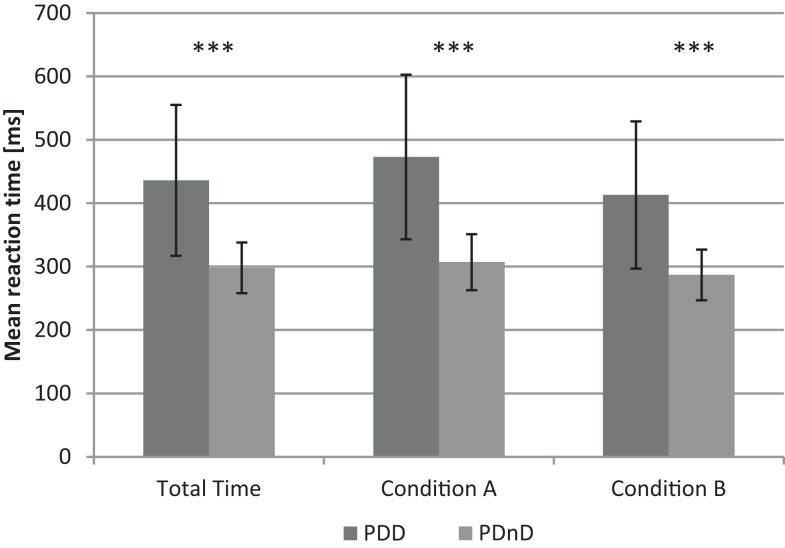
**Mean reaction time (ms) for total time, condition A (without acoustic cue) and condition B (with application of acoustic cue) in PDD and the PDnD group**.

Patients with PDD showed a significantly prolonged reaction time in all four experimental blocks: PDD: Mm = 436 ms (SD = 119 ms); PDnD: Mm = 298 ms (40 ms; *p* < 0.001). This effect was independent from presenting [blocks 2 and 3; PDD: Mm = 413 ms (116 ms); PDnD: Mm = 287 ms (39 ms); *p* < 0.001] or not presenting [blocks 1 and 4; PDD: Mm = 473 ms (130 ms): PDnD: Mm = 307 ms (SD = 43 ms); *p* < 0.001] the additional acoustic cue. An overview of the results is given in Figure 1.

### Reaction to the acoustic cue

**Figure 2:**
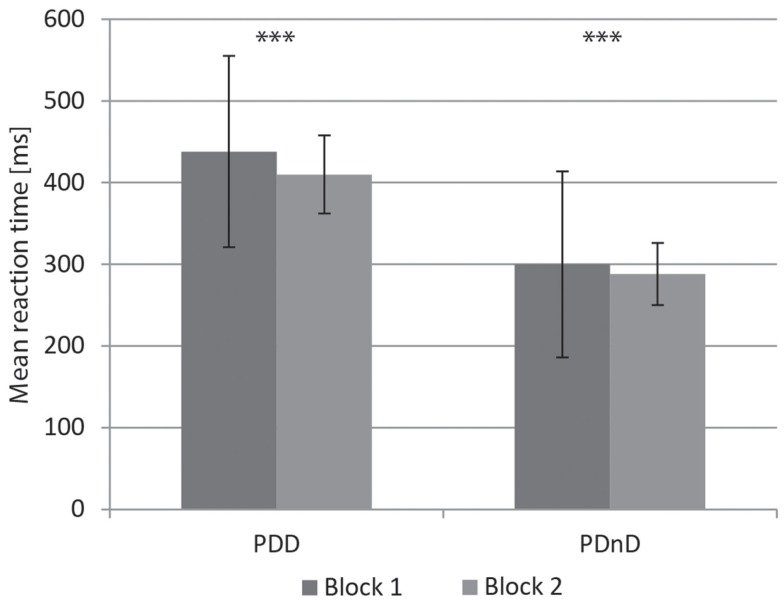
**Reaction to the acoustic cue: mean reaction times (ms) for block 1 and block 2 in the PDD and the PDnD group**.

**Figure 3:**
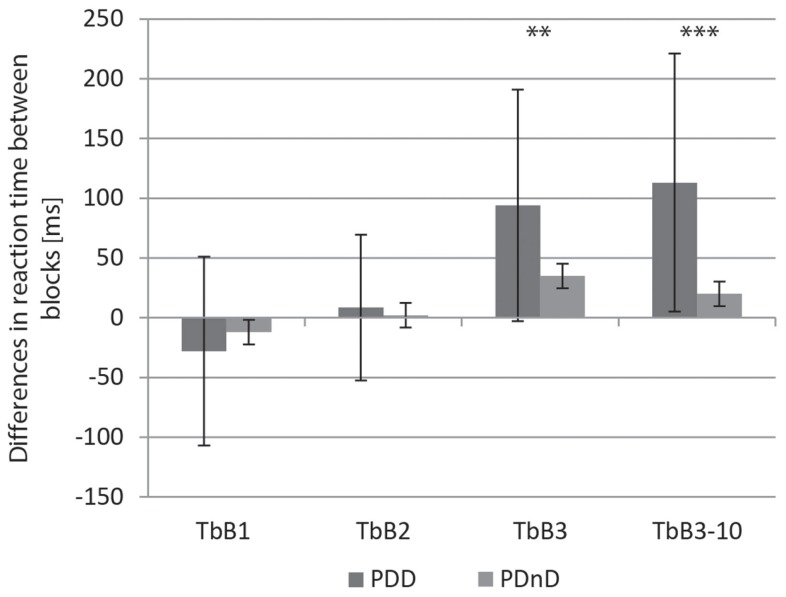
**Transition effects between the respective test blocks [transition between blocks (TbB)] for the three transitions using all trials (TbB1–TbB3) and additionally for the third transition using the last 10 trials of block 3 and the first 10 trails of block 4 (“TbB3–10”) for the PDD and the PDnD group, differences in reaction time between blocks (ms)**.

Both groups (PDD and PDnD) reduced their reaction time under cue presentation and showed accelerated response from block 1 to block 2 [PDD: 438 ms (117 ms) versus 410 ms (114 ms), *p* < 0.001; PDnD: 300 ms (48 ms) versus 288 ms (38 ms), *p* < 0.001, Figure 2]. The results are shown in Table 2. Analysis of the transition between the respective blocks (see Figure 3) according to the TbB values revealed no significant group differences between PDD and PDnD patients for TbB1 (*p* = 0.485) and TbB2 (*p* = 0.480). Both patient groups showed comparable transition effects between the respective test blocks.

**Table 2:** **Transition effects between the respective test blocks [transition between blocks (TbB)] for the three transitions using all trials (TbB1–TbB3) and additionally for the third transition using the last 10 trials of block 3 and the first 10 trails of block 4 (“TbB3–10”) for the PDD and the PDnD group; means of medians (and standard deviations) in ms**.

	PDD	PDnD	*p*-value
TbB1	−28 ms (79 ms)	−12 ms (32 ms)	0.397
TbB2	9 ms (61 ms)	2 ms (31 ms)	0.806
TbB3	94 ms (97 ms)	35 ms (44 ms)	0.028
TbB3-10	113 ms (108 ms)	20 ms (44 ms)	0.001

### Withdrawal from acoustic cue

After the withdrawal of the acoustic cue, a significant increase in reaction time was observable in both experimental groups from block 3 to block 4 [PDD: 451 ms (152 ms) versus 552 ms (171 ms), *p* < 0.001; PDnD: 308 ms (51 ms) versus 345 ms (54 ms), *p* < 0.001]. In contrast to the comparable transition effects in TbB1 and TbB2, the TbB3 values (between blocks 3 and 4) were significantly higher in the PDD group than in the PDnD group (*p* = 0.009, see Table 2; Figure 3). This indicates that PDD patients might have more problems to maintain their performance in the visual reaction time task after withdrawal of the acoustic cue (condition A).

### *Post hoc* analysis

The *post hoc* analysis of TbB using only the 10 first trials of block 4 (first half of block 4) and the last 10 trials of block 3 revealed an even larger difference, attaining a highly significant level between blocks 3 and 4 (*p* < 0.001, see Table 2; Figure 3). This shows that especially in the first half of block 4, a prolongation of reaction time occurs for the PDD group.

### Testing for systematic variability on reaction time within blocks

The comparison of the first five trials with the last five trials of each block revealed. There is no significant effect indicating a progression in reaction time shortening in blocks 1, 2, 3, and 4 (*p* = 0.586, *p* = 0.460, *p* = 0.141, and *p* = 0.232; see results in Table 3). There is no evidence for a shortening in reaction time due to the administration of a series of trails within the same block. Effects observed in block 4 cannot, therefore, be explained by a general speed up of reaction time and are depending on the cognitive status. This is even demonstrated in the significant group effect (*p* = 0.005; see Table 3), showing a clear difference between the first five trails and the last five trails only in the PDD group (*post hoc* pairwise comparison of groups: *p* < 0.001).

**Table 3:** **Results of ANOVA on reaction time within blocks**.

	Md Trial 1–5 Mean (SD) in ms	Md Trail 16–20 Mean (SD) in ms	*p*-value	*p*-value main factor group (PDD, PDnD)
Block 1	385.96 (134.4)	351.34 (101.87)	*p* = 0.586	*p* = 0.731
Block 2	3554.42 (120.08)	365.98 (131.63)	*p* = 0.460	*p* = 0.323
Block 3	361.38 (137.21)	360.64 (126.26)	*p* = 0.141	*p* = 0.337
Block 4	441.68 (196.79)	400.44 (117.32)	*p* = 0.232	*p* = 0.005

## Discussion

Purpose of the study was to investigate the effect of global cognitive deterioration as seen in dementia on the usage of an acoustic cue in a SRT paradigm in PD. There is increasing evidence that such cueing strategies can improve motor performance in particular in everyday life situations (25). However, this cannot be generalized since cuing can also lead to attention shift, distraction, and additional stress simply because of the introduction of an additional task (14, 15). So far, these aspects have not been well investigated in PDD. Moreover, in this study, we also put a particular focus on the period after the withdrawal of the cue because of its everyday clinical relevance.

We observed a significant reduction of reaction time in both experimental groups (PDD and PDnD) after introduction of the cue. Interestingly, the improvement of reaction time was comparable between the PDD and PDnD groups. Cognitive decline thus does not play a role in primarily using the stimulus to speed up reaction time. In other words, the cueing processes seem to be unaffected by cognitive deterioration due to the dementing process. However, our study also showed that the removal of the cue leads to longer reaction times in PDD patients.

The SRT paradigm used in this study has the advantage that it investigates the presentation and the withdrawal of a cue at once. In addition, the task uses a simple motor response reducing the load on patients who already suffer from impaired motor functions.

A closer look at the pathomechanism of hypokinesia may help to understand the beneficial effect of cueing in PDD and its adverse effect in other types of dementia. In recent work with animal models of PD, changes in network activity have been described in detail and an increase in the power of the beta-band (13–35 Hz) oscillations within the motor cortex has been observed and has been associated with hypokinesia (26, 27). Furthermore, deep brain stimulation in PD patients reduces hypokinesia by overriding the beta-oscillations (28), probably by partly desynchronizing the beta-oscillations (29). A hypothesis that could explain our finding is that the sensory stimulus of the external cue could act on the neuronal population by desynchronizing the beta-band oscillations. This hypothesis would require intact long-range cortico-cortical connections, which have been implicated in beta-band oscillations (30). In patients suffering from Alzheimer’s dementia, beta-band oscillations are generally reduced in power (31). The general assumption is that this is due to the loss of long distance connections (32). Our data show that PDD patients still benefit from external cuing and therefore we could assume that the influence of the external stimulus on the beta-band neuronal activity is still present. This could then indicate that in PDD – in contrast to Alzheimer’s dementia – the long-range connections are intact and that other cortical mechanisms contribute to the cognitive decline of PDD patients. This view fits with the observation that different cortical pathologies are involved in PDD and Alzheimer’s dementia (33). A prediction of this view would be that in PDD patients beta-band oscillations would be similar to those seen in PDnD; however, further studies are required to investigate this issue.

An additional important finding of this study was the observation that the removal of the cue resulted in a prolongation of the reaction time in PDD patients, which differed significantly from that of PDnD patients. One interpretation is that the PDD group was substantially more irritated from the removal of the cue than the non-demented individuals. As PDD patients showed readjustment of the reaction time in the second half of block 4 (see Figure 3; Table 2), it seems unlikely that this effect was caused by fatigue. By controlling for other confounding factors like age, disease duration, or motor function, only the overall cognitive situation of the PDD group remains as the only reasonable explanatory factor. Another hypothesis could be that the sudden withdrawal of the stimulus in PDD patients did not provide for adequate time for the resetting of central processes to baseline activity.

The paradigm of cueing is used in many non-pharmacological therapeutic interventions, first of all in therapy of gait (25, 34, 35). Based on our results, cues can, therefore, also be considered for cognitively impaired PD patients. However, it has to be taken into account that the achieved advantage by use of a cue can be at the expense of a deterioration of reaction time directly after the withdrawal of the cue. Thus, permanent application of cues might be an appropriate solution in such patients. Similar findings were reported by Lim et al. (34, 35) after cued gait training in PD. After the intervention period, their patients did not show a stable training effect without the presence of the cue and the achieved effect disappeared. Although the authors excluded patients with a Mini-Mental State Examination score <25 (34, 35), it is – based on our results – intriguing to hypothesize that the effect observed by the authors might be explained by the inclusion of PD patients with at least slight cognitive impairment. The authors discussed that a permanent cueing device may be a useful option to overcome this problem; however, continuous cueing may have other downsides, such as adaptation.

In line with these data, we suggest that the cognitive state of PD patients is responsible for the sustainability of the cueing effect. The identification of this principle, i.e., that the achieved gain has a high probability to disappear in PDD patients, could open new ways to find better treatment strategies. The therapeutic use of cueing mechanism seems tempting in PDD patients as this method is easy to apply in the patient’s environment with low costs, but certainly demands new and individualized training programs. Our data give rise to the assumption that it might be reasonable to allocate different patient groups according to their cognitive level to different training programs to get more efficient therapy outcomes. However, this has to be verified in further studies.

Our study faces some limitations, since we only used simple finger movements in order to minimize the motor impact. As daily-relevant movements are generally more complex, our data must be confirmed in future studies using more complex paradigms. Furthermore, we did not include PD patients with mild cognitive impairment (19), which is considered a transition state between normal and severely impaired cognition. The investigation of such a cohort would help to better understand when the above deficits occur in the course of cognitive decline in PD.

In summary, our data show that cortical and BG involvement as seen in PDD does not affect patients’ ability to benefit from external cue presentation. Therefore, pathomechanisms associated with this phenomenon seem to be unaffected by cognitive impairment in PD. However, the observation that the withdrawal of the acoustic stimulus causes prolonged reaction times in PDD patients shows a clear involvement of general cognitive worsening and has to be taken into consideration when cueing strategies are applied also to PDD.

## Conflict of Interest Statement

The authors declare that the research was conducted in the absence of any commercial or financial relationships that could be construed as a potential conflict of interest.
